# Setting the Game Agenda: Reviewing the Emerging Literature on Video Gaming and Psychological Well-Being of Sexual and Gender Diverse Youth

**DOI:** 10.1177/15554120231178883

**Published:** 2023-06-08

**Authors:** Dane Marco Di Cesare, Shelley L. Craig, Ashley S. Brooks, Kaitrin Doll

**Affiliations:** 1 Faculty of Education, 7497 Brock University, Sr. Catharines, Ontario, Canada; 2 Factor-Inwentash Faculty of Social Work, 152790 University of Toronto, Toronto, Ontario, Canada

**Keywords:** sexual and gender diverse youth, video games, digital microaggressions, digital communities, well-being

## Abstract

Video gaming is a popular youth pastime that has prompted scholarship into its relationship with psychological well-being. However, sexual and gender diverse youth (SGDY) who play video games are largely overlooked in this research. SGDY experience significant mental health challenges, but utilize coping strategies mediated by digital technologies, necessitating an examination of their video game playing and its effects on well-being. This literature review synthesizes the emerging evidence base by identifying key constructs related to SGDY well-being and video gaming. Five themes were derived from the literature: (a) SGDY identity development and self-expression in video games; (b) SGDY video gaming and coping skills; (c) Social support in SGDY video gaming communities; (d) SGDY digital microaggressions in video gaming; and (e) SGDY civic engagement through video gaming. The findings establish multiple risks and opportunities for harnessing video games to support SGDY's well-being. Recommendations for practice, research, and industry collaborations are presented.

## Introduction

Video games are a mainstream, interactive, and immersive technology-based form of entertainment that is rapidly replacing music as the most important aspect of youth culture today (Monahan, 2021). Western reports on youth technology trends use “youth” as a broad term to collectively describe several developmental periods lasting roughly from early adolescence to early adulthood. Nearly 78% of U.S. households with youth aged 8–18 own a gaming console (Rideout & Robb, 2019), and 90% of Canadian adolescents (aged 13–17) actively play video games (Entertainment Software Association of Canada, 2020). Australian households with children rank video games as the third most popular media choice, above music, reading, and social media (Brand et al., 2017); and video gaming ranks fourth as the most popular activities for young people aged 8–25 in the United Kingdom (Nominet, 2022). Video games span various genres, such as combat-focused first-person shooters and collaborative massively multiplayer online roleplaying games (MMORPGs), which can typically be discerned by their unique design conventions, stories, and themes.

Research on the social and psychological impacts of video gaming has changed over time in response to the aging demographic of video game players, the growing diversity of video game players, increasing complexity and diversity of video games, and their growing entrenchment in the culture (Dale & Shawn Green, 2017). Scholarship on video gaming accelerated in the early 2000s, with common topics of interest being the impacts of video gaming on attention, dexterity, aggression, and sedentary behaviors (Anderson et al., 2004). Alongside these research areas, video game research also focuses on the links between video gaming and well-being, and how to harness the popularity and design of video games to enhance players’ professional, educational, and health outcomes (Micallef et al., 2022).

### Video Gaming and Well-being

Well-being is an overall positive psychological state combining several factors such as autonomy, environmental mastery, personal growth, positive relationships with others, a sense of purpose, and self-acceptance (Ryff, 2013). Evidence for multiple positive and negative affective, cognitive, and behavioral impacts on well-being related to video games has been presented in the literature. Video games can promote enhanced emotional regulation through immersive flow-like states during fun gameplay, mastery, and appreciation of its fictional properties (Villani et al., 2018); social connection through collaborative play (Raith et al., 2021); and the fostering of an overall optimistic motivational style (Granic et al., 2014). While there is some evidence that playing violent video games contributes to aggressive affect and decreased empathy and prosociality (Anderson et al., 2010), it has been difficult to replicate these findings (Ferguson, 2020). Research has also documented associations between video game playing and improved top-down attention and spatial awareness (Bediou et al., 2018); working memory (Waris et al., 2019); and executive control (Li et al., 2020) but there is also some evidence that playing video games during adolescence can be related to decreases in attention at school (Swing et al., 2010).

Given that there may be affective and cognitive benefits of video gaming (e.g., entertainment), players may be motivated to play to support their well-being (Johannes et al., 2021). Video games played through physical exercise may also support the physical well-being of players by promoting fitness and activeness (Zeng & Gao, 2016). However, video gaming has also been linked to several dysfunctional behaviors among problematic video game players (i.e., gamers who meet most of the criteria for video game addiction) who comprised 8% of the sample in one study of 1,178 U.S. youth aged 8–18 (Gentile, 2009). Estimates from surveys in Norway and Germany further suggest that 0.2–0.4% report addictive video game use, meeting all of the criteria for video game addiction (Festl et al., 2013; Mentzoni et al., 2011). Problematic and addictive video gaming is negatively associated with well-being and predicts higher rates of depression and anxiety symptoms and sleep disturbance (Männikkö et al., 2020). Relatedly, video games may feature paid luck-based features resembling gambling (e.g., mystery loot boxes), which have been linked to higher rates of problem gambling and spending in a large survey of 7,427 U.S. adult gamers (Zendle & Cairns, 2019).

While much has been learned about the effects of video gaming on well-being, most of the research has studied the general population of video game players, overlooking demographic differences. For example, problematic and addictive video gaming may be more prevalent among boys and young men experiencing loneliness (Pallavicini et al., 2022). Further investigation of marginalized video game players is particularly warranted as evidence indicates that online video gaming can be a vehicle for racist, sexist, and ableist abuse by other players (Cote, 2017; Crooks & Magnet, 2018; TaeHyuk Keum & Hearns, 2022).

### Digital Risks and Resilience Among Sexual and Gender Diverse Youth

Recent research highlights that sexual and gender diverse youth (SGDY) can live drastically different offline and online lives. Offline, SGDY can experience familial rejection (Salerno et al., 2022), adverse childhood experiences (Craig et al., 2020), discrimination from healthcare providers (Henriquez & Ahmad, 2021), and homophobic and transphobic bullying at school (Domínguez-Martínez & Robles, 2019; Moyano & Sánchez-Fuentes, 2020). In turn, these minority stressors (Meyer, 2003) can increase the risk of developing mental illnesses like anxiety and depression and problematic behaviors like substance use, self-harm, and suicide (Russell & Fish, 2016).

While these negative experiences could make SGDY more susceptible to problematic and addictive gaming behaviors and psychological distress from online harassment (Arcelus et al., 2017; Gillin & Signorella, 2023), other findings suggest that SGDY often live vibrant and fulfilling digital lives that are protective for SGDY experiencing harm offline (Craig et al.,^ 2015^; Craig & McInroy, 2014). The internet is a vital source of information and resources for SGDY, which can support identity development and service use (McInroy, McClosky et al., 2020). SGDY have also been found to be creating affirmative virtual peer support network on platforms such as YouTube, Facebook, and Tumblr, which provide respite from stigma and violence, belonging, confidence, hope, and political mobilization (Craig et al., 2020, 2021; Tortajada et al., 2021).

Altogether, these behaviors may constitute a “digital resilience”; digital processes and actions that generate positive growth (Craig et al., 2023) but, to date, much of the research in this area has focused on social media, online streaming services, forums, and websites. Given that trans people and gay males aged 14–29 have particularly high rates of video game console ownership within SGDY populations, video games could be a critical but overlooked site that contributes to the well-being of SGDY (McInroy, Craig et al., 2019). Indeed, approximately 80% of U.S. teens aged 13–17 own a video game console, with even greater numbers owning desktop computers/laptops (90%) and smartphones (95%) with gaming capabilities (Pew Research Center, 2022). As such, the present literature review aims to collate the research findings in this emergent area and answer the following research question: *How does video game playing relate to the well-being of SGDY?*

## Methods

Creswell and Creswell (2018) propose the following literature search recommendations: (1) identifying search terms that capture the research topic's core domains; (2) inputting search terms into research databases; (3) prioritizing peer-reviewed journal articles and incorporating other published and unpublished materials as appropriate; (4) ascertaining relevance of the found articles; (5) grouping relevant articles thematically; (6) summarizing key articles; and (7) writing the results.

Search terms utilizing Boolean operators were used, such as “sexual and gender diverse,” “LGBT*” and its constituent identity labels, “youth,” “young adults,” “well*,” “mental*,” “resilience,” “video gam*,” names of specific video game franchises, and “Gaymer,” a label that some SGD video game players adopt for themselves (Sundén, 2009). The search terms were entered into research databases (e.g., Google Scholar, JSTOR, Semantic Scholar) and abstracts from peer-reviewed English-language papers published since 2000 were screened for relevance to the research question and the target age cohort of 10–29 years. Accepted articles were read thoroughly and grouped thematically according to their findings. Where research themes required further development and context, findings from book chapters, unpublished graduate theses, gray literature, and journalism were incorporated. Finally, the findings were synthesized and key research gaps and opportunities for future research were identified.

## Results

The literature review collated 27 peer-reviewed journal articles, four book chapters, three news articles, three conference proceedings, two reports, and one Master's thesis. The literature was grouped into five themes: (a) SGDY identity development and self-expression in video games; (b) SGDY video gaming and stress coping skills; (c) Social support through SGD video gaming communities; (d) SGD digital microaggressions in video gaming; and (e) SGDY civic engagement through video gaming.

### SGDY Identity Development and Self-Expression in Video Games

One of the most widely studied aspects of SGD identities and video game playing is the avatar, an in-game representation of the player whose name and appearance can be customized (Morningstar & Farmer, 2008). In one focus group study of 17 trans and gender diverse youth aged 11–22, the participants reported that they used avatars to explore, develop, and rehearse their gender identities and expressions, supporting their well-being (Morgan et al., 2020). Another interview-based study with 10 trans and gender diverse youth aged 13–18 found that avatar customization facilitated participants to explore their gender in a low-stakes environment, affirming their feelings and decisions about their evolving gender identity, signaling to other players how they wish to be gendered, and feeling closer to an aspirational gendered self (J. L. McKenna et al., 2022). Specifically, choosing a name, pronouns, and a personalized look for the avatar provided an agentic way to cue other players and the video game itself how they wanted to be seen and addressed. Accordingly, other players and dialogue in the game respond to these gender signifiers at face value, affirming the player's gender. This may be particularly beneficial for younger trans and gender diverse youth exploring their identity.

It has been theorized that roleplaying as one's avatar can reduce uncomfortable feelings of perceived discrepancy between the actual self (i.e., the person as they currently are) and the ideal self (i.e., the person as they would like to be) because it allows one to emulate and act out aspirational aspects of the ideal self (Klimmt et al., 2010). In gameplay, this can enhance the player's enjoyment in the game and temporarily alter the player's self-concept, facilitating more positive feelings toward oneself (Klimmt et al., 2009). One published case study documents the author's innovative attempt to integrate the avatar into a therapeutic setting with a trans patient struggling with their gender identity and presentation, which appeared to support the building of rapport (Rivera, 2022). Although embodying a gender-affirming avatar may have a positive psychological influence on the player (Strauss et al., 2019), these feelings are temporary and state bound to the in-game world (Klimmt et al., 2010). As such, the potential benefits of an affirming in-game avatar may be inhibited by important offline factors such as poor mental health, familial rejection, and other interpersonal problems (i.e., discrimination and transphobia), requiring further study (Arcelus et al., 2017).

There is also some growing interest in how SGD video game streamers (i.e., people who broadcast themselves playing games on platforms such as Twitch) construct and manage their identities while livestreaming video games. In a qualitative study of 25 Twitch streamers (eight of whom identified as SGD), participants indicated that sharing their sexual and gender diversity with their audience was a common coming out “ritual” that may also encourage viewers to come out in their offline lives (Freeman & Wohn, 2020). Twitch streaming offers several technical and practical affordances that facilitate this identity management. For trans and gender diverse youth who are uncomfortable with broadcasting their present appearance or voice, technologies such as greenscreen or experimenting with camera positioning allow them to exert control over how they are seen by their audience to mitigate against online harassment (Freeman & Wohn, 2020).

In summary, research highlights that video games and video game streaming offer several technical affordances that support the self-expression, agency, and autonomy of SGDY, and especially that of trans and gender diverse youth. Video game avatar creation specifically seems to play a notable role in identity exploration, expression, and validation among trans and gender diverse youth who play video games. Emerging evidence suggests that player avatars could be harnessed in therapeutic contexts, but further study is needed.

### SGDY Video Gaming and Stress Coping Skills

Video games provide a highly entertaining experience through their storylines, visual aesthetics, gameplay, music and sound design, and cultural significance, and video games with SGDY representation may be especially appreciated by SGDY players who do not see their lives reflected in media (Kohlburn et al., 2023). Given the enjoyment and fun derived from video games, video gaming has been associated with a range of coping skills among SGDY, varying in adaptiveness. In a survey of 711 trans and gender diverse youth aged 14–25 in Australia, 57.7% reported using video games to make them feel better when experiencing distress, whereas 54% reported accessing mental health support/counseling and 40.2% reported accessing peer support groups or youth centers (Strauss et al., 2017). A semistructured interview study with 20 SGDY aged 14–18 who were experiencing bullying elaborates that playing video games was useful for “sidetracking” (e.g., coping through distraction; Craig et al., 2020) and that video games provided a highly customizable environment that allowed them to assert agency and autonomy, reducing stress and other negative feelings (R. T. O’Brien et al., 2022). These findings are extended in a semistructured interview study with 12 trans and gender diverse adults aged 21–34 who discussed their experiences playing choice-based games (in which the storyline is driven by choices that the player makes). The findings highlighted that the high degree of fun, autonomy, customization, and freedom within these games instilled a sense of meaningfulness into the players’ choices and created a low-stakes environment to explore and make sense of oneself through inclusive character creation and dating features (Cantrell & Zhu, 2022).

Nonetheless, some evidence suggests that excessive video gaming is detrimental to SGDY well-being. In one qualitative study of self-care practices during COVID-19 shelter-in-place orders using open-ended cross-sectional survey responses of 770 sexual minority adolescents aged 15–19, several participants indicated that they played video games to escape reality and connect with their peers whom they could not see in-person. However, some participants communicated that excessive video gaming for escapism may have negative mental health consequences and isolate them from their peers (O’Brien et al., 2021). While video gaming may have facilitated coping processes (i.e., distraction, escapism, agency) that reduced psychological distress and supported the well-being of SGDY during COVID, more research is needed to ascertain when these coping strategies are maladaptive and the psychosocial factors that predict problematic video gaming behaviors among SGDY (Arcelus et al., 2017; Broman & Hakansson, 2018).

A complementary area of scholarship is exploring the potential applications of serious video games in evidence-based therapeutic interventions for SGDY. Video game-based mental health interventions may demonstrate a high degree of acceptability among SGDY because therapeutic approaches can be discreetly woven into the game narrative and mechanics while preserving aspects of video games (e.g., customization) that already appeal to the target audience (Strauss et al., 2019). One such serious game, SPARX, is a computerized cognitive behavioral therapy self-help program available for free for youth in New Zealand and shows some promise in reducing depressive symptoms among adolescents (Merry et al., 2012). An adaptation for sexual diverse adolescents, Rainbow SPARX, demonstrated similarly positive results (Lucassen et al., 2015). However, SPARX presently lacks the affordances of many commercial video games (e.g., gender expansive character design options), which undermines its acceptability among trans and gender diverse youth specifically (Lucassen et al., 2021). Co-design to refine SPARX with trans and gender diverse youth has indicated that factors like binary character creation options and a lack of bespoke SGD content are key participation barriers for this cohort (Lucassen et al., 2018; Strauss et al., 2019).

Overall, there is consistent evidence showing that SGDY play video games for entertainment, distraction, and escapism, which can support stress coping and well-being. However, there is also recognition that this manner of coping may be less adaptive in the face of chronic stressors requiring diverse coping skills and may be problematic or addictive among SGDY experiencing greater psychosocial risk or younger SGDY who are still developing stress coping skills. Yet, there is also promising emerging evidence that SGDY can develop more adaptive coping skills through a video game-based mental health intervention, yet more research is needed to improve its acceptability among this cohort and further build the evidence base for its efficacy in improving key indicators of well-being.

### Social Support Through SGD Gaming Communities

Research findings show that online communities can be vital sources of support for SGDY (Austin et al., 2020; McInroy, 2020). However, much of the research to date has focused on fan communities based on TV and film, with relatively less attention to how SGDY connect around their shared affinity for video gaming (Shaw, 2010). Social media offers ample opportunities for SGD gamers to connect with each other around their shared interest in video games on platforms such as Discord in SGD texting and audiovisual chat servers, on Reddit in SGD Subreddits such as r/Gaymers and r/TransGamers, and by watching SGD video game streamers and interacting with other audience members on Twitch (Brown & Moberly, 2020; Fiorellini, 2021).

Research on SGD video gamer subcultures has also studied player-made communities in MMORPGs, which allow players to create and roleplay as their own characters, immerse themselves in a rich storyline, improve skills, and play with up to millions of other players online (Romero, 2017). Among the most popular MMORPG titles today are Final Fantasy XIV (FFXIV) and World of Warcraft (WoW), which are each estimated to be played by over 2 million people daily (MMO Populations, n.d.). A focus group study with 14 trans and gender nonconforming youth aged 11–18 found that MMORPGs were a highly accessible place for finding SGD peers internationally and provided consistent sources of support, information, social networks, and therapeutic experiences (Strauss et al., 2019). Another systematic review of 18 peer-reviewed studies showed that playing MMORPGs may improve self-esteem, reduce feelings of loneliness and depression, and promote a positive outlook on life (Raith et al., 2021). Accordingly, MMORPGs are a popular digital space for community building, with one survey of 30,000 MMORPG players estimating that 79% join collaborative (and sometimes competitive) social groups variously known as “guilds,” “clans,” “free companies,” and other terms (Yee, 2006), which are often integrated with text-based, audiovisual, and teleconferencing platforms like Discord. For clarity, these communities will be collectively referred to as *guilds*.

Early SGD video game guilds were founded in response to hostile and microaggressive video gaming communities (Kelley, 2012). Developers have disciplined guild founders for advertising alleged “exclusionary” SGD guilds (Shaw, 2015) and analysis of MMORPG forums have also echoed these hostilities within the player base (Pulos, 2013). As such, SGD guilds enforce rules that prohibit hate speech and preserve a positive atmosphere (Collister, 2014). SGD guilds are particularly attractive for players living in rural and conservative areas to reliably have fun and foster friendship, highlighting important motivational parallels in help-seeking behaviors within hostile online and offline ecosystems (McKenna & Chughtai, 2020).

To summarize, video games are a common interest among SGDY, around which communities with a predominantly SGD membership form. These communities aim to maintain an affirming and positive environment, enacted through rules and moderation, and include social media-based sites on Reddit as well as communities specific to certain video game titles and franchises. MMORPGs are particularly well documented as a virtual space for SGDY to find inclusive and accepting communities, potentially related to their tendency to include comprehensive avatar creation features—a key technical affordance for SGDY players.

### SGD Digital Microaggressions While Video Gaming

Digital spaces can expose marginalized users to messages and content that can jeopardize their well-being (Keighley, 2022; Ștefăniță & Buf, 2021). One survey of U.S. adult gamers aged 18–45 (*n*  =  1045) found that 74% of online multiplayer gamers reported an experience of harassment in-game, with 35% of SGD players reporting harassment based on gender identity or sexual orientation (Ingersoll, 2020). Research has documented that heterosexual video game players engage in cyberbullying more than do SGD players and, inversely, that SGD players experience significantly higher rates of sexually related cyber victimization (Ballard & Welch, 2017). These digital microaggressions, while often brief, communicate hostile and derogatory views about SGD and other marginalized people (Nadal et al., 2016) and are pervasive online (Craig et al., 2023), threatening the well-being of younger SGDY who may not have yet developed adaptive coping skills (Craig et al., 2020). Racialized players also report being unfairly targeted in player-versus-player combat when they are identified by other players as being a person of color (Cote, 2017; Gray, 2012, 2018; Nakamura, 2012). Less cooperative in-game activities that rely on skill, individualism, and competitiveness (e.g., player-versus-player combat; PvP) are less likely to motivate positive interpersonal relationships with other players (Castillo, 2019). However, there is not yet any documented evidence of similarly biased in-game violence against players identified as (or perceived to be) SGD.

Gamers from marginalized communities can be targets of “hate raids” (Thach et al., 2024), coordinated attacks organized to spam hate messages facilitated by the anonymity of the internet and automated bot software that is able to rapidly communicate hate speech through text messages and text to speech (Uttarapong et al., 2021). The moderation responses of gaming-related social media platforms may also contribute to a microaggressive digital environment for SGDY. A policy analysis of Twitch's content rules prohibiting obscene and pornographic content suggests that they could be applied in biased ways that discriminate against SGD video game streamers (Ruberg, 2021). As well, trans and gender diverse youth may face unique challenges in engaging with voice and video communications as they may be uncomfortable about how they look or sound and be misgendered and harassed by others (Baldwin, 2018).

Video game microaggressions may also be communicated in environmental ways that subtly invalidate and erase sexual and gender diversity and other intersectional experiences. Video game mechanics such as gender-restricted clothing limit the ability of SGD players to express and experiment with their gender presentation and identity, alienating gender diverse players (McKenna et al., 2022; Morgan et al., 2020). Similarly, although same-sex relationships and romance options are increasingly present in video games (Shaw et al., 2019; Utsch et al., 2017), they have also been critiqued for emulating heteronormative values and not reflecting more transgressive aspects of SGD identity and relationships (Greer, 2013). SGD characters may also embody negative stereotypes like being a sexual predator or psychotic (Colliver, 2020). As well, MMORPGs have been criticized for their conspicuous absence of Black and other people of color, further impacting racialized SGDY (Gray, 2012).

Overall, there is strong evidence that digital microaggressions are commonly enacted against SGDY in video games and video game communities. Despite the unique and diverse ways (e.g., in-game violence) video games, players, and gaming-related social media platforms can communicate microaggressions that exclude and stigmatize SGDY players, there is little peer-reviewed literature studying the possible impacts this could have on the well-being of SGDY. While some research indicates that SGDY may develop and strengthen coping skills (e.g., help-seeking, reframing, utilizing platform features like blocking) when exposed to hateful social media content (Craig et al., 2020), this has not been adequately studied in the context of video gaming accounting for developmental differences between younger and older SGDY.

### SGDY Civic Engagement Through Video Gaming

A notable extension of the findings from the prior two themes is the civic engagement of SGD gamers in response to discrimination, microaggressions, and violence. Responses from a survey of U.S. adolescents (*n*  =  1102) identified that those who played games with civic experiences, defined as simulations of civic or political activities (e.g., helping other players, debating ethical issues), were more likely to express interest in politics or current events, and were more likely to give or raise money for charity (Granic et al., 2014; Lenhart et al., 2020). As such these players may be more civically and politically engaged, engaging in self-advocacy behaviors such as protesting, persuading others to vote in an election, and staying informed about current events (Lenhart et al., 2008).

Participating in politically engaged guilds may enable SGD to influence political or social justice movements through virtual protest. For example, one ethnographic study of an SGD social movement within WoW forums found that SGD players banded together online to address reporting procedures for anti-SGD hate in-game, unifying against harassment, hate speech, and digital microaggressions. Player-led campaigns also targeted WoW's developer to implement change, leading to the revision of reporting procedures and the removal of discriminatory policies that placed restrictions on the use of SGD acronyms for avatars (McKenna & Chughtai, 2020). Similarly, in response to inadequate content moderation practices, in 2021, marginalized Twitch streamers protested. This action resulted in Twitch adding new account verification options and suing two hate raid participants who persistently targeted marginalized streamers with racist, sexist, and homophobic content (Chalk, 2022; Thach et al., 2024). Organizing has also taken the form of community mourning; in response to the traumatic community losses in the Pulse shooting in Orlando on June 12, 2016, FFXIV players held a public vigil to honor the victims and process their grief (Mother Vane, 2016).

In summary, there is anecdotal evidence that political engagement and self-advocacy do occur within video games and video game communities. The use of social media to “fight back” against oppression and develop leadership skills has been observed among SGDY elsewhere (Craig et al., 2020, 2023) but there has so far been no research exploring how self-advocacy in video gaming may support SGDY in developing leadership and coping skills (e.g., assertiveness) that may support their well-being.

## Discussion

The literature review presented five emerging themes outlining the potential effects that video game playing may have on the well-being of SGDY. Evidently, video games can be a vital source of social connection and support for SGD players; afford customization and features that allow SGDY to control their gaming experience, explore and express themselves, and protect themselves online; and provide some ways of coping with adversity and minority stress. However, significant challenges include antisocial and hate speech from other players, inadequate SGD representation in video game content, the limited adaptiveness of coping skills developed through video gaming, problematic gaming behaviors, and the limited application of these findings to consumer video gaming. Altogether, these early findings suggest that video games could represent an innovative and technology-engaged way to support the resilience of SGDY, but more research is required.

### A Digital World of Opportunities

Recent research findings indicate that SGDY use social media to support their well-being by way of emotional regulation, identity development, self-advocacy, and community building (Craig et al., 2023). The emerging findings reviewed here indicate that SGDY are similarly using video games to support their well-being, but research has so far made limited efforts to operationalize and measure well-being quantitatively. As part of a repertoire of coping strategies, video gaming may be helpful in mitigating against minority stressors in both physical and digital worlds by providing entertainment, escapism, cathartic experiences, supportive communities, space to explore oneself, resonant SGD storylines, and personal growth. However, these constructs require explicit measurement and testing.

These findings would have important implications for researchers, community members, and industry who all stand to gain from the creation of more positive, safe, and enriching online experiences for marginalized and vulnerable SGDY who are entrenched in video game subculture (Strauss et al., 2017). Further, creating safer spaces may attract more SGDY to gaming. Efforts are also underway to develop evidence-based mental health interventions in the form of serious games (Lucassen et al., 2015, 2021, 2022) and future research should seek to understand how to best tailor these interventions to the diverse needs of SGDY, how to best integrate therapeutic approaches into video game design, and how they can be designed in innovative ways that are responsive to the increasing availability of consumer technologies like virtual reality (Bolesnikov et al., 2022).

### The Need for Academic-Industry Alliance

The findings point toward significant risks on platforms but addressing these challenges requires working closely with the video game and social media industries to ensure that their technologies contain features, safeguarding, themes, characters, and storylines that preserve and nurture the well-being of SGDY and other marginalized communities while also providing an entertaining experience (Kohlburn et al., 2023). Trends toward more SGD representation in video games over time and a gradual queering of the game developer profession may indicate some industry receptiveness toward addressing social issues in video games (Shaw et al., 2019). However, much of this work is being taken up by SGD video game enthusiasts creating modifications (or “mods”) as a hobby (Howard, 2021) or by indie developers (an individual or small team of game developers without monetary or technical support from a larger game publisher) with smaller audiences (Ruberg, 2019), pointing toward a need for more uptake among mainstream developers and popular franchises. This work could expand and diversify video game audiences; encourage creativity and innovation; enhance the reputation of video gaming; and promote positive social change. Relatedly, while there is promising evidence of the efficacy of serious games in supporting SGDY well-being, these video games are unable to engage players longitudinally because they are highly structured and self-contained in scope. As such, there are significant opportunities for academic-industry partnerships to investigate how mainstream video games can create more prosocial play and support the well-being of players beyond entertainment (Snodgrass et al., 2020).

Another significant area for improvement that the research highlights is content moderation and community building in online multiplayer games, whose in-game chats can be used to bully, abuse, and harass marginalized players (Kou & Gui, 2021). Current video game community moderation is evidently not sufficient in safeguarding vulnerable SGDY and other marginalized players—and may even censor them (Thach et al., 2024). Recent structural equation modeling suggests that teamwork, competition, player competence, and player embodiment of and identification with their in-game avatars are all key constructs that can influence the likelihood of espousing prejudicial views while playing video games (Cary et al., 2020). Academic-industry collaboration may be able to uncover how these factors can be manipulated in video games to promote prosocial play and player well-being.

### Limitations

There are some limitations to highlight. While many of the findings speak favorably to the potential benefits of video gaming on SGDY well-being, most of the studies are qualitative. Although there is methodological diversity, encompassing semistructured interviews, focus groups, digital ethnography, screenshot elicitation, and text-based interviews, their small sample sizes limit their generalizability. As well, much of the findings are couched within the lens of media and cultural studies, queer studies, communications, video game studies, and human–computer interaction research. More research from the health sciences that adopts a stronger developmental lens is required, given how broadly “youth” is defined in the literature. As well, because the search was restricted to English-language papers, much of the collated literature pertained to Western research contexts. Further, the search incorporated a small selection of book chapters, news articles, conference proceedings, reports, and a graduate thesis to supplement the peer-reviewed journal article findings. Altogether, these limitations highlight a need for more quantitative research, disciplinary buy-in from health sciences and applied social sciences researchers, and more robust studies accounting for developmental and cultural differences.

## Conclusion

This literature review showed that video games may contribute to the well-being of SGDY in both positive and negative ways. Video games may support facets of SGDY well-being such as stress coping skills, identity development, self-expression, creativity, skill mastery, affirming community, and civic engagement. But video gaming can also expose SGDY to hate speech and digital microaggressions or develop into a maladaptive coping behavior. Research now needs to quantitatively test these key constructs to elaborate on and generalize emerging qualitative findings and more fully understand video gaming as a coping behavior among SGDY. This scholarship would be of value to researchers and practitioners working in mental health, video game industry professionals, who can work toward making video gaming a safer and more inclusive hobby, and SGDY video gamers who will ultimately benefit from this work.

## References

[bibr1-15554120231178883] Anderson C. A. Funk J. B. Griffiths M. D. (2004). Contemporary issues in adolescent video game playing: Brief overview and introduction to the special issue. Journal of Adolescence, 27(1), 1–3. 10.1016/j.adolescence.2003.10.001

[bibr2-15554120231178883] Anderson C. A. Shibuya A. Ihori N. Swing E. L. Bushman B. J. Sakamoto A. Rothstein H. R. Saleem M. (2010). Violent video game effects on aggression, empathy, and prosocial behaviour in Eastern and Western countries: A meta-analytic review. Psychological Bulletin, 136(2), 151–173. 10.1037/a0018251 20192553

[bibr3-15554120231178883] Arcelus J. Bouman W. P. Jones B. A. Richards C. Jimenez-Murcia S. Griffiths M. D. (2017). Video gaming and gaming addiction in transgender people: An exploratory study. Journal of Behavioral Addictions, 6(1), 21–29. 10.1556/2006.6.2017.002 28198637 PMC5572994

[bibr4-15554120231178883] Austin A. Craig S. L. Navega N. McInroy L. B. (2020). It’s my safe space: The life-saving role of the internet in the lives of transgender and gender diverse youth. International Journal of Transgender Health, 21(1), 33–44. 10.1080/15532739.2019.1700202 33015657 PMC7430466

[bibr5-15554120231178883] Baldwin K. (2018). Virtual avatars: Trans experiences of ideal selves through gaming. Markets, Globalization & Development Review, 3(3), Article 4. 10.23860/mgdr-2018-03-03-04

[bibr6-15554120231178883] Ballard M. E. Welch K. M. (2017). Virtual warfare: Cyberbullying and cyber-victimization in MMOG play. Games and Culture, 12(5), 466–491. 10.1177/1555412015592473

[bibr7-15554120231178883] Bediou B. Adams D. M. Mayer R. E. Tipton E. Green C. S. Bavelier D. (2018). Meta-analysis of action video game impact on perceptual, attentional, and cognitive skills. Psychological Bulletin, 144(1), 77–110. 10.1037/bul0000130.supp 29172564

[bibr8-15554120231178883] Bolesnikov A. Golshan A. Tierney L. Mann A. Kang J. Girouard A. (2022). Queering e-therapy: Considerations for the delivery of virtual reality based mental health solutions with LGBTQ2IA+ communities. In Lewy H. Barkan R. (Eds.), Pervasive computing technologies for healthcare (pp. 183–202). Springer.

[bibr9-15554120231178883] Brand J. E. Todhunter S. Jervis J. (2017). Digital Australia 2018. IGEA. https://igea.net/2017/07/digital-australia-2018-da18/

[bibr10-15554120231178883] Broman N. Hakansson A. (2018). Problematic gaming and internet use but not gambling may be overrepresented in sexual minorities - A pilot population web survey study. Frontiers in Psychology, 9, Article 2184. 10.3389/fpsyg.2018.02184 PMC6243046 30483191

[bibr11-15554120231178883] Brown A. M. L. Moberly L. (2020). Twitch and participatory cultures. In Kowert R. Quandt T. (Eds), The video game debate 2: Revisiting the physical, social, and psychological effects of video games (pp. 53–65). Routledge. 10.4324/9780429351815

[bibr12-15554120231178883] Cantrell Y. Zhu X. A. (2022). Choice-based games and resilience building of gender nonconforming individuals: A phenomenological study. Digital Transformation and Society, 1(2), 198–218. 10.1108/dts-08-2022-0039

[bibr13-15554120231178883] Cary L. A. Axt J. Chasteen A. L. (2020). The interplay of individual differences, norms, and group identification in predicting prejudiced behavior in online video game interactions. Journal of Applied Social Psychology, 50(11), 623–637. 10.1111/jasp.12700

[bibr14-15554120231178883] Castillo R. P. (2019). Exploring the differential effects of social and individualistic gameplay motivations on bridging social capital for users of a massively multiplayer online game. Computers in Human Behavior, 91, 263–270. 10.1016/j.chb.2018.10.016

[bibr15-15554120231178883] Chalk A. (2022, March 14). *More hate raids strike Twitch as white supremacist takes credit*. PC Gamer. https://www.pcgamer.com/more-hate-raids-strike-twitch-as-white-supremacist-takes-credit/

[bibr16-15554120231178883] Collister L. B. (2014). Surveillance and community: Language policing and empowerment in a World of Warcraft guild. Surveillance & Society, 12(3), 337–348. 10.24908/ss.v12i3.4956

[bibr17-15554120231178883] Colliver B. (2020). Representation of LGBTQ communities in the Grand Theft Auto series. In Kelly C. Lynes A. Hoffin K. (Eds.), Video games crime and next-gen deviance (pp. 131–149). Emerald Publishing Limited.

[bibr18-15554120231178883] Cote A. C. (2017). “I can defend myself”: Women’s strategies for coping with harassment while gaming online. Games and Culture, 12(2), 136–155. 10.1177/1555412015587603

[bibr19-15554120231178883] Craig S. L. Austin A. Levenson J. Leung V. W. Y. Eaton A. D. D'Souza S. A. (2020). Frequencies and patterns of adverse childhood events in LGBTQ+ youth. Child Abuse & Neglect, 107, Article 104623. 10.1016/j.chiabu.2020.104623 32682145

[bibr20-15554120231178883] Craig S. L. Brooks A. S. Doll K. Eaton A. D. McInroy L. B. Hui J. (2023). Processes and manifestations of digital resilience: Video and textual insights from sexual and gender minority youth. Journal of Adolescent Research, Advance Online Publication. 10.1177/07435584221144958 PMC11828692 39958466

[bibr21-15554120231178883] Craig S. L. Eaton A. D. Kirkland A. Egag E. Pascoe R. King K. Krishnan S. (2021). Towards an integrative self: A digital photo elicitation study of resilience among key marginalized populations of sexual and gender minority youth. International Journal of Qualitative Studies on Health and Well-Being, 16(1), Article 1961572. 10.1080/17482631.2021.1961572 PMC8366624 34375157

[bibr22-15554120231178883] Craig S. L. McInroy L. B. (2014). You can form a part of yourself online: The influence of new media on identity development and coming out for LGBTQ youth. Journal of Gay and Lesbian Mental Health, 18(1), 95–109. 10.1080/19359705.2013.777007

[bibr23-15554120231178883] Craig S. L. McInroy L. B. McCready L. T. Di Cesare D. M. Pettaway L. (2015). Connecting without fear: Clinical implications of the consumption of information and communication technologies by sexual minority youth and young adults. Clinical Social Work Journal, 43(2), 159–168. 10.1007/s10615-014-0505-2

[bibr24-15554120231178883] Creswell J. W. Creswell D. J. (2018). Research design: Qualitative*,* quantitative, and mixed methods approaches (5th ed.). SAGE.

[bibr25-15554120231178883] Crooks H. R. Magnet S. (2018). Contests for meaning: Ableist rhetoric in video games backlash culture. Disability Studies Quarterly, 38(4). 10.18061/dsq.v38i4.5991

[bibr26-15554120231178883] Dale G. Shawn Green C. (2017). The changing face of video games and video gamers: Future directions in the scientific study of video game play and cognitive performance. Journal of Cognitive Enhancement, 1(3), 280–294. 10.1007/s41465-017-0015-6

[bibr27-15554120231178883] Domínguez-Martínez T. Robles R. (2019). Preventing transphobic bullying and promoting inclusive educational environments: Literature review and implementing recommendations. Archives of Medical Research, 50(8), 543–555. 10.1016/j.arcmed.2019.10.009 32036103

[bibr28-15554120231178883] Entertainment Software Association of Canada (2020). *Real Canadian gamer essential facts* 2020. https://essentialfacts2020.ca/

[bibr29-15554120231178883] Ferguson C. J. (2020). Aggressive video games research emerges from its replication crisis (sort of). Current Opinion in Psychology, 36, 1–6. 10.1016/j.copsyc.2020.01.002 32146151

[bibr30-15554120231178883] Festl R. Scharkow M. Quandt T. (2013). Problematic computer game use among adolescents, younger and older adults. Addiction, 108(3), 592–599. 10.1111/add.12016 23078146

[bibr31-15554120231178883] Fiorellini N. (2021). *Venn diagram of LGBTQ* *+* *and gaming communities goes here*. JSTOR Daily. https://daily.jstor.org/venn-diagram-of-lgbtq-and-gaming-communities-goes-here/

[bibr32-15554120231178883] Freeman G. Wohn D. Y. (2020). Streaming your identity: Navigating the presentation of gender and sexuality through live streaming. Computer Supported Cooperative Work: CSCW: An International Journal, 29(6), 795–825. 10.1007/s10606-020-09386-w

[bibr33-15554120231178883] Gentile D. (2009). Pathological video-game use among youth ages 8 to 18. Psychological Science, 20(5), 594–602. 10.1111/j.1467-9280.2009.02340.x 19476590

[bibr34-15554120231178883] Gillin L. E. Signorella M. L. (2023). Attitudes toward sexual orientation and gender identity in online multiplayer gaming spaces. Psychological Reports. Advance online publication. 1–23. 10.1177/00332941231153798 PMC11529123 36688329

[bibr35-15554120231178883] Granic I. Lobel A. Engels R. C. M. E. (2014). The benefits of playing video games. American Psychologist, 69(1), 66–78. 10.1037/a0034857 24295515

[bibr36-15554120231178883] Gray K. L. (2012). Intersecting oppressions and online communities: Examining the experiences of women of color in Xbox Live. Information Communication and Society, 15(3), 411–428. 10.1080/1369118X.2011.642401

[bibr37-15554120231178883] Gray K. L. (2018). Gaming out online: Black lesbian identity development and community building in Xbox Live. Journal of Lesbian Studies, 22(3), 282–296. 10.1080/10894160.2018.1384293 29166214

[bibr38-15554120231178883] Greer S. (2013). Playing queer: Affordances for sexuality in fable and dragon age. Journal of Gaming and Virtual Worlds, 5(1), 3–21. 10.1386/jgvw.5.1.3_1

[bibr39-15554120231178883] Henriquez N. R. Ahmad N. (2021). “The message is you don’t exist”: Exploring lived experiences of rural lesbian, gay, bisexual, transgender, queer/questioning (LGBTQ) people utilizing health care services. SAGE Open Nursing, 7, 1–10. 10.1177/23779608211051174 PMC8642108 34869861

[bibr40-15554120231178883] Howard K. T. (2021). Queerness and modification in mainstream and indie games: Examining problems with queer representation in video games and exploring design solutions. *HT 2021 - Proceedings of the 32nd ACM Conference on Hypertext and Social Media*, 101–109. 10.1145/3465336.3475114

[bibr41-15554120231178883] Ingersoll C. (2020). *Free to play?: Hate, harassment and positive social experiences in online games.* https://www.adl.org/resources/report/free-play-hate-harassment-and-positive-social-experience-online-games-2020

[bibr42-15554120231178883] Johannes N. Vuorre M. Przybylski A. D. (2021). Video game play is positively correlated with well-being. Royal Society Open Science, 8(2), Article 202049. 10.1098/rsos.202049 PMC8074794 33972879

[bibr43-15554120231178883] Keighley R. (2022). Hate hurts: Exploring the impact of online hate on LGBTQ + young people. Women and Criminal Justice, 32(1–2), 29–48. 10.1080/08974454.2021.1988034

[bibr44-15554120231178883] Kelley J. (2012). Gay naming in online gaming. Names: A Journal of Onomastics, 60(4), 193–200. 10.1179/0027773812Z.00000000030

[bibr45-15554120231178883] Klimmt C. Hefner D. Vorderer P. (2009). The video game experience as “true” identification: A theory of enjoyable alterations of players’ self-perception. Communication Theory, 19(4), 351–373. 10.1111/j.1468-2885.2009.01347.x

[bibr46-15554120231178883] Klimmt C. Hefner D. Vorderer P. Roth C. Blake C. (2010). Identification with video game characters as automatic shift of self-perceptions. Media Psychology, 13(4), 323–338. 10.1080/15213269.2010.524911

[bibr47-15554120231178883] Kohlburn J. Cho H. Moore H. (2023). Players’ perceptions of sexuality and gender-inclusive video games a pragmatic content analysis of Steam reviews. Convergence: The International Journal of Research into New Media Technologies, 29(2), 379–399. 10.1177/13548565221137481

[bibr48-15554120231178883] Kou Y. Gui X. (2021). Flag and flaggability in automated moderation: The case of reporting toxic behavior in an online game community. *Conference on Human Factors in Computing Systems – Proceedings*, 1–12. 10.1145/3411764.3445279

[bibr49-15554120231178883] Lenhart A. Kahne J. Middaugh E. Macgill A. R. Evans C. Vitak J . (2008). *Teens, video games, and civics: Teen's gaming experiences are diverse and include significant social interaction and civic engagement* . Pew Internet and American Life Project. https://eric.ed.gov/?id=ED525058

[bibr50-15554120231178883] Li X. Huang L. Li B. Wang H. Han C. (2020). Time for a true display of skill: Top players in League of Legends have better executive control. Acta Psychologica, 204, Article 103007. 10.1016/j.actpsy.2020.103007 32000064

[bibr51-15554120231178883] Lucassen M. F. G. Merry S. N. Hatcher S. Frampton C. M. A. (2015). Rainbow SPARX: A novel approach to addressing depression in sexual minority youth. Cognitive and Behavioral Practice, 22(2), 203–216. 10.1016/j.cbpra.2013.12.008

[bibr52-15554120231178883] Lucassen M. F. G. Samra R. Iacovides I. Fleming T. Shepherd M. Stasiak K. Wallace L. (2018). How LGBT + young people use the internet in relation to their mental health and envisage the use of e-therapy: Exploratory study. JMIR Serious Games, 6(4), e11249. 10.2196/11249 PMC6320432 30578194

[bibr53-15554120231178883] Lucassen M. F. G. Samra R. Rimes K. A. Brown K. E. Wallace L. M. (2022). Promoting resilience and well-being through co-design (The PRIDE Project): Protocol for the development and preliminary evaluation of a prototype resilience-based intervention for sexual and gender minority youth. JMIR Research Protocols, 11(2), e31036. 10.2196/31036 PMC8848231 35103613

[bibr54-15554120231178883] Lucassen M. F. G. Stasiak K. Fleming T. Frampton C. Perry Y. Shepherd M. Merry S. N. (2021). Computerized cognitive behavioural therapy for gender minority adolescents: Analysis of the real-world implementation of SPARX in New Zealand. Australian and New Zealand Journal of Psychiatry, 55(9), 874–882. 10.1177/0004867420976846 33287554 PMC8404718

[bibr55-15554120231178883] Männikkö N. Ruotsalainen H. Miettunen J. Pontes H. M. Kääriäinen M. (2020). Problematic gaming behaviour and health-related outcomes: A systematic review and meta-analysis. Journal of Health Psychology, 25(1), 67–81. 10.1177/1359105317740414 29192524

[bibr56-15554120231178883] McInroy L. B. (2020). Building connections and slaying basilisks: Fostering support, resilience, and positive adjustment for sexual and gender minority youth in online fandom communities. Information, Communication & Society, 23(13), 1874–1891. 10.1080/1369118X.2019.1623902

[bibr57-15554120231178883] McInroy L. B. Craig S. L. Leung V. W. Y. (2019). Platforms and patterns for practice: LGBTQ+ Youths’ use of information and communication technologies. Child and Adolescent Social Work Journal, 36(5), 507–520. 10.1007/s10560-018-0577-x

[bibr58-15554120231178883] McInroy L. B. McCloskey R. J. Craig S. L. Eaton A. D. (2019). LGBTQ+ youths’ community engagement and resource seeking online versus offline. Journal of Technology in Human Services, 37(4), 315–333. 10.1080/15228835.2019.1617823

[bibr59-15554120231178883] McKenna B. Chughtai H. (2020). Resistance and sexuality in virtual worlds: An LGBT perspective. Computers in Human Behavior, 105, Article 106199. 10.1016/j.chb.2019.106199

[bibr60-15554120231178883] McKenna J. L. Wang Y.-C. Williams C. R. McGregor K. Boskey E. R. (2022). “You can’t be deadnamed in a video game”: Transgender and gender diverse adolescents’ use of video game avatar creation for gender-affirmation and exploration. Journal of LGBT Youth, 1–21. Advance online publication. 10.1080/19361653.2022.2144583

[bibr61-15554120231178883] Mentzoni R. A. Brunborg G. S. Molde H. Myrseth H. Skouverøe K. J. M. Hetland J. Pallesen S. (2011). Problematic video game use: Estimated prevalence and associations with mental and physical health. Cyberpsychology, Behavior, and Social Networking, 14(10), 591–596. 10.1089/cyber.2010.0260 21342010

[bibr62-15554120231178883] Merry S. N. Stasiak K. Shepherd M. Frampton C. Fleming T. Lucassen M. F. G. (2012). The effectiveness of SPARX, a computerised self help intervention for adolescents seeking help for depression: Randomised controlled non-inferiority trial. BMJ (Online), 344, e2598. 10.1136/bmj.e2598 PMC3330131 22517917

[bibr63-15554120231178883] Meyer I. H. (2003). Prejudice, social stress, and mental health in lesbian, gay, and bisexual populations: Conceptual issues and research evidence. Psychological Bulletin, 129(5), 674–697. 10.1037/0033-2909.129.5.674 12956539 PMC2072932

[bibr64-15554120231178883] Micallef D. Parker L. Brennan L. Schivinski B. Jackson M. (2022). Improving the health of emerging adult gamers—a scoping review of influences. Nutrients, 14(11), Article 2226. 10.3390/nu14112226 PMC9182998 35684027

[bibr65-15554120231178883] MMO Populations (n.d.). *MMO Populations*. https://mmo-population.com/top/2023

[bibr66-15554120231178883] Monahan S. (2021). Video games have replaced music as the most important aspect of youth culture. https://www.theguardian.com/commentisfree/2021/jan/11/video-games-music-youth-culture

[bibr67-15554120231178883] Morgan H. O’donovan A. Almeida R. Lin A. Perry Y. (2020). The role of the avatar in gaming for trans and gender diverse young people. International Journal of Environmental Research and Public Health, 17(22), 1–11. 10.3390/ijerph17228617 PMC7699515 33233536

[bibr68-15554120231178883] Morningstar C. Farmer F. R. (2008). The lessons of Lucasfilm’s Habitat. Journal For Virtual Worlds Research, 1(1), 1–21. 10.4101/jvwr.v1i1.287

[bibr69-15554120231178883] Mother Vane (2016). *FFXIV - Vigil for LGBT Orlando Pulse massacre*. https://www.youtube.com/watch?v=RdItmDAOLtk&ab_channel=MotherVain

[bibr70-15554120231178883] Moyano N. Sánchez-Fuentes M. D. M. (2020). Homophobic bullying at schools: A systematic review of research, prevalence, school-related predictors and consequences. Aggression and Violent Behavior, 53, Article 101441. 10.1016/j.avb.2020.101441

[bibr71-15554120231178883] Nadal K. L. Whitman C. N. Davis L. S. Erazo T. Davidoff K. C. (2016). Microaggressions toward lesbian, gay, bisexual, transgender, queer, and genderqueer people: A review of the literature. Journal of Sex Research, 53(4-5), 488–508. 10.1080/00224499.2016.1142495 26966779

[bibr72-15554120231178883] Nakamura L. (2012). “It’s a n***** in here! Kill the n*****!”: User-generated media campaigns against racism, sexism, and homophobia in digital games. In Valdivia A. N. Nerone J. C. Mayer V. Mazzarella S. R. Parameswaran R. E. Scharrer E. Darling-Wolf F. Gates K. (Eds.), The international encyclopedia of media studies (pp. 2–15). Wiley-Blackwell.

[bibr73-15554120231178883] Nominet (2022). Digital youth index report 2022. https://digitalyouthindex.uk/

[bibr74-15554120231178883] O’Brien R. P. Parra L. A. Cederbaum J. A. (2021). “Trying my best”: Sexual minority adolescents’ self-care during the COVID-19 pandemic. Journal of Adolescent Health, 68(6), 1053–1058. 10.1016/j.jadohealth.2021.03.013 PMC8154726 33875330

[bibr75-15554120231178883] O’Brien R. T. Gagnon K. W. Egan J. E. Coulter R. W. S. (2022). Gaming preferences and motivations among bullied sexual and gender minority youth: An interview study. Games for Health Journal, 11(2), 79–84. https://doi.org/10.1089/g4https://doi.org/ h.2021.0059 35049380 10.1089/g4h.2021.0059 PMC9057884

[bibr76-15554120231178883] Pallavicini F. Pepe A. Mantovani F. (2022). The effects of playing video games on stress, anxiety, depression, loneliness, and gaming disorder during the early stages of the COVID-19 pandemic: PRISMA systematic review. Cyberpsychology, Behavior, and Social Networking, 25(6), 334–354. 10.1089/cyber.2021.0252 35639118

[bibr77-15554120231178883] Pew Research Center (2022). Teens, social media and technology 2022. https://www.pewresearch.org/internet/wp-content/uploads/sites/9/2022/08/PI_2022.08.10_Teens-and-Tech_FINAL.pdf

[bibr78-15554120231178883] Pulos A. (2013). Confronting heteronormativity in online games: A critical discourse analysis of LGBTQ sexuality in world of warcraft. Games and Culture, 8(2), 77–97. 10.1177/1555412013478688

[bibr79-15554120231178883] Raith L. Bignill J. Stavropoulos V. Millear P. Allen A. Stallman H. M. Mason J. de Regt T. Wood A. Kannis-Dymand L. (2021). Massively multiplayer online games and well-being: A systematic literature review. Frontiers in Psychology, 12, Article 698799. 10.3389/fpsyg.2021.698799 PMC8277937 34276523

[bibr80-15554120231178883] Rideout V. Robb M. B. (2019). *The Common Sense census: Media use by tweens and teens.* https://www.commonsensemedia.org/research/the-common-sense-census-media-use-by-tweens-and-teens-2019

[bibr81-15554120231178883] Rivera S. (2022). From battleground to playground: The video game avatar as transitional phenomenon for a transgender patient. Journal of the American Psychoanalytic Association, 70(3), 485–510. 10.1177/00030651221104487 35938569

[bibr82-15554120231178883] Romero S. G. (2017). *Queering space: LGBTQ* gaming as a form of resistance and community development* [Unpublished master’s thesis]. Ryerson University.

[bibr83-15554120231178883] Ruberg B. (2019). The precarious labor of queer indie game-making: Who benefits from making video games “better”? Television and New Media, 20(8), 778–788. 10.1177/1527476419851090

[bibr84-15554120231178883] Ruberg B. (2021). “Obscene, pornographic, or otherwise objectionable”: Biased definitions of sexual content in video game live streaming. New Media and Society, 23(6), 1681–1699. 10.1177/1461444820920759

[bibr85-15554120231178883] Russell S. T. Fish J. N. (2016). Mental health in lesbian, gay, bisexual, and transgender (LGBT) youth. Annual Review of Clinical Psychology, 12(1), 465–487. 10.1146/annurev-clinpsy-021815-093153 PMC4887282 26772206

[bibr86-15554120231178883] Ryff C. D. (2013). Psychological well-being revisited: Advances in the science and practice of eudaimonia. Psychotherapy and Psychosomatics, 83(1), 10–28. 10.1159/000353263 24281296 PMC4241300

[bibr87-15554120231178883] Salerno J. P. Gattamorta K. A. Williams N. D. (2022). Impact of family rejection and racism on sexual and gender minority stress among LGBTQ+ young people of colour during COVID-19. Psychological Trauma: Theory, Research, Practice, and Policy, 15(4), 637–647. https://psycnet.apa.org/doi/10.1037/tra0001254 . 10.1037/tra0001254 35511543 PMC10361835

[bibr88-15554120231178883] Shaw A. (2010). What is video game culture? Cultural studies and game studies. Games and Culture, 5(4), 403–424. https://psycnet.apa.org/doi/10.1177/1555412009360414 . 10.1177/1555412009360414

[bibr89-15554120231178883] Shaw A. (2015). Talking to gaymers: Questioning identity, community and media representation. Westminster Papers in Communication and Culture, 9(1), 67–89. 10.16997/wpcc.150

[bibr90-15554120231178883] Shaw A. Lauteria E. W. Yang H. Persaud C. J. Cole A. M. Leigh Waldron E. Freisem E. West N. Thach K. Phillips A. Ruberg B. Nguyen J. Agloro A. (2019). Counting queerness in games: Trends in LGBTQ digital game representation, 1985-2005. International Journal of Communication, 13(26), 1544–1569. https://ijoc.org/index.php/ijoc/article/view/9754

[bibr91-15554120231178883] Snodgrass J. G. Clements K. R. Nixon W. C. Ortega C. Lauth S. Anderson M. (2020). An iterative approach to qualitative data analysis: Using theme, cultural models, and content analyses to discover and confirm a grounded theory of how gaming inculcates resilience. Field Methods, 32(4), 399–415. 10.1177/1525822X20939749

[bibr92-15554120231178883] Ștefăniță O. Buf D. (2021). Hate speech in social media and its effects on the LGBT community: A review of the current research. Romanian Journal of Communication and Public Relations, 23(1), 47–55. 10.21018/rjcpr.2021.1.322

[bibr93-15554120231178883] Strauss P. Cook A. Winter S. Watson V. Toussaint D. W. Lin A. (2017). *Trans pathways: The mental health experiences and care pathways of trans young people.* https://www.telethonkids.org.au/projects/past/trans-pathways/

[bibr94-15554120231178883] Strauss P. Morgan H. Wright Toussaint D. Lin A. Winter S. Perry Y. (2019). Trans and gender diverse young people’s attitudes towards game-based digital mental health interventions: A qualitative investigation. Internet Interventions, 18, Article 100280. 10.1016/j.invent.2019.100280 PMC6926275 31890628

[bibr95-15554120231178883] Sundén J. (2009). Play as transgression: An ethnographic approach to queer game cultures. Breaking new ground: Innovation in games, play, practice and theory. *Proceedings of the Digital Games Research Association (DiGRA) Digital Games Research Conference.* https://citeseerx.ist.psu.edu/document?repid=rep1&type=pdf&doi=5261df66d49cb8f68b29e2836016e55bc5ae2ed1

[bibr96-15554120231178883] Swing E. L. Gentile D. A. Anderson C. A. Walsh D. A. (2010). Television and video game exposure and the development of attention problems. Pediatrics, 126(2), 214–221. 10.1542/peds.2009-1508 20603258

[bibr97-15554120231178883] TaeHyuk Keum B. Hearns M. (2022). Online gaming and racism: Impact on psychological distress among Black, Asian, and Latinx emerging adults. Games and Culture, 17(3), 445–460. 10.1177/15554120211039082

[bibr98-15554120231178883] Thach H. Mayworm S. Delmonaco D. Haimson O. (2024). (In)visible moderation: A digital ethnography of marginalized users and content moderation on Twitch and Reddit. New Media and Society, 26(7), 4034–4055. 10.1177/14614448221109804

[bibr99-15554120231178883] Tortajada I. Willem C. Platero Méndez R. L. Araüna N. (2021). Lost in Transition? Digital trans activism on Youtube. Information, Communication & Society, 24(8), 1091–1107. 10.1080/1369118X.2020.1797850

[bibr100-15554120231178883] Utsch S. Bragança L. C. Ramos P. Caldeira P. Tenorio J. (2017). Queer identities in video games: Data visualization for a quantitative analysis of representation. *SBC – Proceedings of SBGames* 2017, 847-854. https://www.sbgames.org/sbgames2017/papers/CulturaFull/175360.pdf

[bibr101-15554120231178883] Uttarapong J. Cai J. Wohn D. Y. Y. (2021). Harassment experiences of women and LGBTQ live streamers and how they handled negativity. *IMX 2021 - Proceedings of the 2021 ACM International Conference on Interactive Media Experiences*, 7–19. 10.1145/3452918.3458794

[bibr102-15554120231178883] Villani D. Carissoli C. Triberti S. Marchetti A. Gilli G. Riva G. (2018). Videogames for emotion regulation: A systematic review. Games for Health Journal, 7(2), 85–99. 10.1089/g4h.2017.0108 29424555

[bibr103-15554120231178883] Waris O. Jaeggi S. M. Seitz A. R. Lehtonen M. Soveri A. Lukasik K. M. Söderström U. Cohen Hoffing R. A. Laine M. (2019). Video gaming and working memory: A large-scale cross-sectional correlative study. Computers in Human Behavior, 97, 94–103. 10.1016/j.chb.2019.03.005 31447496 PMC6707529

[bibr104-15554120231178883] Yee N. (2006). The demographics, motivations, and derived experiences of users of massively multi-user online graphical environments. Presence: Teleoperators and Virtual Environments, 15(3), 309–329. 10.1162/pres.15.3.309

[bibr105-15554120231178883] Zendle D. Cairns P. (2019). Video game loot boxes are linked to problem gambling: Results of a large-scale survey. PLoS ONE, 14(3), e0214167. 10.1371/journal.pone.0214167 PMC6417717 30870502

[bibr106-15554120231178883] Zeng N. Gao Z. (2016). Exergaming and obesity in youth: Current perspectives. International Journal of General Medicine, 9, 275–284. https://doi.org/10.2147%2FIJGM.S99025 27536158 10.2147/IJGM.S99025 PMC4977069

